# Detection of a CTX-M group 2 beta-lactamase gene in a *Klebsiella pneumoniae* isolate from a tertiary care hospital, Trinidad and Tobago

**DOI:** 10.1186/s12941-017-0209-x

**Published:** 2017-05-08

**Authors:** Paul Cheddie, Francis Dziva, Patrick Eberechi Akpaka

**Affiliations:** 1 Department of Medical Technology, University of Guyana, Georgetown, Guyana; 2 Faculty of Medical Sciences, The University of the West Indies, St. Augustine, Trinidad and Tobago; 3 Department of Para-Clinical Sciences, Faculty of Medical Sciences, The University of the West Indies, St. Augustine, Trinidad and Tobago

**Keywords:** Extended-spectrum beta-lactamase, Polymerase chain reaction, Random amplification of polymorphic DNA, Trinidad and Tobago

## Abstract

**Background:**

Identification of the prevalence and spread of ESBL-mediated antibiotic resistance is essential especially in the hospital setting. It is for this reason, more and more studies are highlighting the importance of complementing phenotypic ESBL-detection techniques with molecular techniques in order to understand the basis and extent of this form of resistance among clinically evolved bacterial populations, especially those belonging to the *Enterobacteriaceae* family. However, in Trinidad and Tobago and other Caribbean countries, very little is known regarding ESBL detection rates and/or the prevalence of genes conferring ESBL resistance.

**Methodology:**

Sixty-six *Klebsiella pneumoniae* isolates from clinical specimens phenotypically identified by the Microscan Walkaway-96 System as potential ESBL-producers were analysed in this study. Screening and confirmation of these isolates as ESBL producers was done by the Clinical and Laboratory Standards Institute (CLSI) approved methods. Polymerase chain reaction amplification of beta-lactamase genes *bla*_TEM_, *bla*_SHV_, *bla*_CTX-M1_, *bla*_CTX-M2_ and *bla*_AmpC_ was performed to identify mechanisms of β-lactam resistance.

**Results:**

ESBL-producing *K. pneumoniae* was confirmed in 78.8% (41/52) from isolates collected from a variety of sources during the period, April–July 2015. *bla*_SHV_ (84.8%) and *bla*_CTX-M_ (46.9%) were the predominant β-lactamase genes identified. A single *K. pneumoniae* isolate possessed a *bla*_CTX-M_ group 2 beta-lactamase gene. RAPD analysis identified a number of epidemiologically related isolates. However, current isolates were unrelated to isolates from previous years.

**Conclusion:**

This study revealed that among *K. pneumoniae* isolates exhibiting extended spectrum β-lactam resistance, there was a high prevalence of *bla*_SHV_ and *bla*_CTX-M_ genes. This result highlights the need for a reliable epidemiological apparatus that involves the molecular characterisation of ESBL resistance.

**Electronic supplementary material:**

The online version of this article (doi:10.1186/s12941-017-0209-x) contains supplementary material, which is available to authorized users.

## Background

Organisms harbouring genes for extended spectrum β-lactamase (ESBL) production are a major public health concern especially given their association with cephalosporin therapy failure, and the burden they place on infection control practices. Since the first reported case in *Klebsiella* isolates in Germany in the late twentieth century [1], they have increasingly been described worldwide, including in the Caribbean [2–4]. ESBLs are β-lactam hydrolysing enzymes capable of conferring bacterial resistance to the penicillins, 1st-, 2nd-, and 3rd-generation cephalosporins, and azetronam (but not the cephamycins or carbapenems), and which are inhibited by β-lactamase inhibitors such as clavulanic acid [5, 6]. The clinical significance of these enzymes is under-pinned by the fact that often times in vitro activity of antimicrobial drugs against ESBL-producing organisms does not always translate into clinical efficacy in patients [7].

Most *Klebsiella pneumoniae* ESBLs are plasmid-encoded enzymes derived classically from the TEM- and SHV- type β-lactamases [1] which belong to molecular class A, according to the classification scheme of Ambler, and Bush–Jacoby–Medeiros 2be group of β-lactamases [5]. TEM and SHV ESBLs are functionally similar to another group of rapidly proliferating β-lactamase enzymes, the CTX-M enzymes, that are related to chromosomally determined β-lactamases in species of *Kluyvera* [8, 9].

Due to the complex epidemiology of ESBL-producing *K. pneumoniae*, the frequency of isolation varies among institutions [10]. Exploring the population diversity of ESBL-harbouring *K. pneumoniae* in a single institution is essential to understanding the role of the genes, plasmids, and clones, involved in ESBL-production, and therapy failure with cephalosporins (and to some extent carbapenems) as well as providing useful information for infection prevention and control initiatives.

The presence and characterization of ESBL-producing genes in clinical isolates of *K. pneumonia*e and other *Enterobacteriaceae* have previously been described in Trinidad and Tobago [2, 3, 11]. However, this study sought to determine the evolving nature of these genes, specifically in the hospital setting, in order to provide data that could be useful in improving infection control measures and guide antimicrobial stewardship programmes.

## Methods

### Setting

The Eric Williams Medical Sciences Complex (EWMSC), a tertiary ambulatory regional hospital in Trinidad and Tobago that provides general healthcare for both paediatric and adult populations.

### Clinical isolates

Seventy-two *K. pneumoniae* isolates were used in this study. This comprised 52 non-duplicate *K. pneumoniae* isolates recovered from the microbiology laboratory during the study period, April–July 2015, and identified as resistant to extended-spectrum β-lactam agents. Additionally, 20 isolates representing a subset recovered in 2008, 2009 and 2010—which were stored at −70 °C in BHI and 5% glycerol and were identified as β-lactamase producers—were also included in this study. Preliminary identification and susceptibility testing of the isolates collected in the current study was determined using the Microscan WalkAway-96 (Beckman Coulter, Inc.). The procedures were performed in accordance with the manufacturer’s recommendations. The breakpoints were interpreted in accordance with Clinical and Laboratory Standards Institute (CLSI) guidelines [12].

### Antimicrobial susceptibility

Extended-spectrum β-lactamase production were confirmed according to the CLSI confirmatory testing guidelines [12]. Briefly, confirmatory testing was performed on Mueller–Hinton agar (BD) using cefotaxime 30 μg, ceftazidime 30 μg, cefotaxime/clavulanic acid 30/10 μg and ceftazidime 30/10 μg (Oxoid, Remel Inc, USA) (Additional file 1). Quality control of the test procedures was performed with *K. pneumoniae* ATCC 700603 and *Escherichia coli* ATCC 25922.

### PCR amplification

From the sum total of isolates analysed (n = 72), 66 (consisting of 57 phenotypically confirmed ESBL producers and 9 non-ESBL producers randomly selected from the pool) were subjected to polymerase chain reaction (PCR) analysis. The non-ESBL producers were included to detect AmpC beta-lactamase production. The PCR amplification of *bla* genes, including *bla*_TEM_, *bla*_SHV_, *bla*_CTX-M-1_, *bla*_CTX-M-2_ and *bla*_AmpC_ were carried out with GoTaq^®^ Green Master Mix (Promega, Madison, Wisconsin) using primers listed in Table 1. All PCR amplicons were separated by gel electrophoresis on a 1.5% (wt/vol) agarose gel. Staining of the gel was conducted with 0.5 μg/ml GelRed™ (Biotium, Hayward, CA).

**Table 1:** Primers used for PCR amplification of *bla* genes

Target	Primer name	Primer sequence (5′–3′)
*bla*_TEM_	TEM-F TEM-R	GCGGAACCCCTATTTG ACCAATGCTTAATCAGTGAG
*bla*_SHV_	SHV-F SHV-R	TTATCTCCCTGTTAGCCACC GATTTGCTGATTTCGCTCGG
*bla*_CTX-M-1_	CTX-M1-F CTX-M1-R	GGTTAAAAAATCACTGCGTC TTGGTGACGATTTTAGCCGC
*bla*_CTX-M-2_	CTX-M2-F CTX-M2-R	GATGAGACCTTCCGTCTGGA CAGAAACCGTGGGTTACGAT
*bla*_*AmpC*_	AmpC-F AmpC-R	ATGATGGGGGGGTCGTTATGC TTGCAGCTTTTCAAGAATGCGC

All primers were obtained from Sigma-Aldrich (St. Louis, MO, USA)

### Random amplification of polymorphic DNA (RAPD) typing

Bacteria were grown overnight on MacConkey agar (Hardy Diagnostics) at 37 °C. Genomic DNA was then extracted using the ChargeSwitch^®^ gDNA Mini Bacteria Kit (Invirogen, Carlsbad, CA) following the specific manufacturer’s instructions. Samples were initially screened for RAPD typing using five different primers: RAPD1 (5′-CGTGGGCCT), RAPD2 (5′-TCGTCGGCGT), RAPD3 (5′-GTGACGTAGG), RAPD4 (5′-CTTGAGTGGA), RAPD5 (5′-GAGATGACGA) (Sigma-Aldrich). RAPD–PCR was conducted under reaction conditions described by Ashayeri-panah et al. [13]. The amplified products were separated by electrophoresis in a 2.0% agarose gel containing GelRed™ run in 1 × TAE buffer at 65 V for 3 h 30 min until amplified fragments are separated. RAPD1 was chosen because it gave the best banding pattern. RAPD typing was then performed using selected isolates (Additional file 1). The resulting gel was photographed under UV light. RAPD fingerprints were analysed with PyElph version 1.4 gel analysis software [14], and a dendrogram generated using the unweighted pair group method with arithmetic averages (UPGMA).

## Results

### Description of isolates and CLSI confirmatory test results

Following initial testing, 52 *K. pneumoniae* isolates were identified during the study period, (April 2015–July 2015), by the Microscan Walkaway-96 as potential ESBL producers. Phenotypic AST confirmatory testing indicated that 41 (78.8%) of these isolates were indeed extended-spectrum β-lactamase producing. Additionally, of the subset of 20 *K. pneumoniae* isolates tested from previous years, 16 (80%) were identified as ESBL-producers after confirmatory testing. Four were *K. pneumoniae* isolated in 2008, nine were isolated in 2009, and three were isolated in 2010.

### Detection and characterisation of *K. pneumoniae* isolates expressing extended-spectrum β-lactamase resistance

Of the 72 *K. pneumoniae* isolates, 66 were examined by PCR to detect the presence of *bla*_TEM_, *bla*_SHV_, *bla*_CTX-M1_, *bla*_CTX-M2_ and *bla*_AmpC_. This comprised the 41 ESBL-confirmed *K. pneumoniae* isolates collected during the study period along with the 16 ESBL-confirmed *K. pneumoniae* isolates collected between 2008 and 2010. Additionally, nine *K. pneumoniae* isolates identified as non-ESBL-producers were chosen randomly and added to the pool. 65 of the 66 isolates possessed a gene that may contribute to β-lactamase production (Table 2). *bla*_TEM_ was detected in 39 of the isolates tested, comprising n = 30 (2015), n = 2 (2008), n = 6 (2009) and n = 1 (2010) *K. pneumoniae* isolates respectively. *bla*_SHV_ was identified in n = 3 (2008), n = 9 (2009), n = 3 (2010), and n = 41(2015) of *K. pneumoniae* isolates respectively. *bla*_CTX-M1_ genes were detected in 30 of the isolates collected in 2015 as well as n = 2 (2008), n = 6 (2009) and n = 2 (2010). Interestingly, 88.9% (8/9) isolates, representing non-ESBL producing isolates that were tested with PCR, were positive for a *bla*_SHV_ gene. Only one *K. pneumoniae* isolate, recovered in 2015, tested positive for a *bla*_CTX-M2_ gene.

**Table 2:** Correlation between ESBL confirmatory results and PCR results

PCR result	Phenotypic confirmatory testing result (Not-confirmed isolates)		Phenotypic confirmatory testing result (Confirmed isolates)	
	Negative (9)	% Total	Positive (57)	% Total
Negative				
bla_TEM_	8	88.8	18	31.6
bla_SHV_	1	11.1	1	1.7
bla_CTX-M1_	8	88.9	17	29.8
bla_CTX-M2_	9	100	56	98.2
bla_AmpC_	9	100	57	100
Positive				
bla_TEM_	1	11.1	39	68.4
bla_SHV_	8	88.9	56	98.2
bla_CTX-M1_	1	11.1	40	70.2
bla_CTX-M2_	0	0	1	1.7
bla_AmpC_	0	100	0	100

Twenty five isolates were positive for *bla*_TEM_, *bla*_SHV_, and *bla*_CTX-M1_ β-lactamase genes representing 21 of the *K. pneumoniae* isolates collected in 2015 in addition to 1 isolate from 2008 and 3 isolates from 2009. Notably, a single isolate collected in 2015 was positive for *bla*_TEM_, *bla*_SHV_, *bla*_CTX-M1_ and *bla*_CTX-M2_ genes.

The majority of the isolates, 80.5% (33/41), that were identified in 2015 as ESBL-producing were retrieved from urine specimens, whilst the remainder were recovered from blood, sputum, wound and genital sources. Records were not available for the 16 ESBL-positive isolates from previous years.

**Fig. 1:**
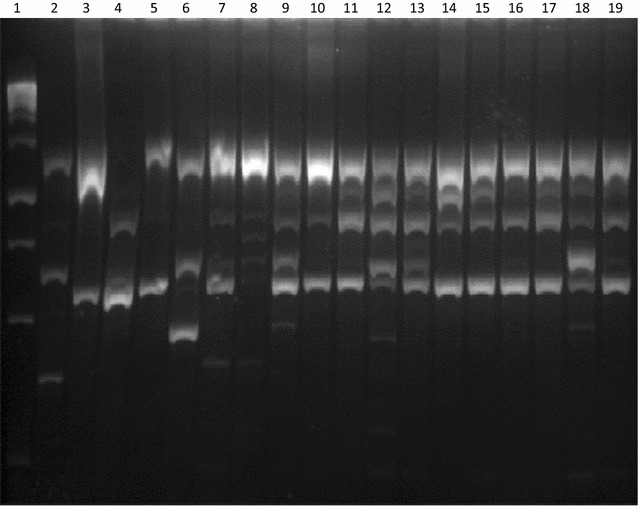
RAPD profiles for a fraction of ESBL-producing *Klebsiella pneumoniae* isolates amplified by RAPD1 primers. From *left* to *right*: GeneRuler 1 kb ladder followed by isolates from 2008 (*lanes 2* and *3*), 2009 (*lanes 4* and *5*), 2010 (*lanes 6*–*8*) and 2015 (*lanes 9*–*19*)

For the generation of DNA fingerprints using RAPD-PCR analysis, 18 isolates were used representing *K. pneumoniae* strains from 2008 to 2010 as well as isolates recovered during the duration of this study (Fig. 1). Banding patterns revealed DNA weights between 250 and 2000 bp (Fig. 1). RAPD analysis revealed that six *K. pneumoniae* isolates had the same banding patterns and these were placed into four genotypic groups. While there were similarities in some of the band positions of the remaining 12 *K. pneumoniae* isolates, their overall RAPD profiles were not similar. Therefore, the conclusion was that these isolates belonged to distinct genotypic groups.

**Fig. 2:**
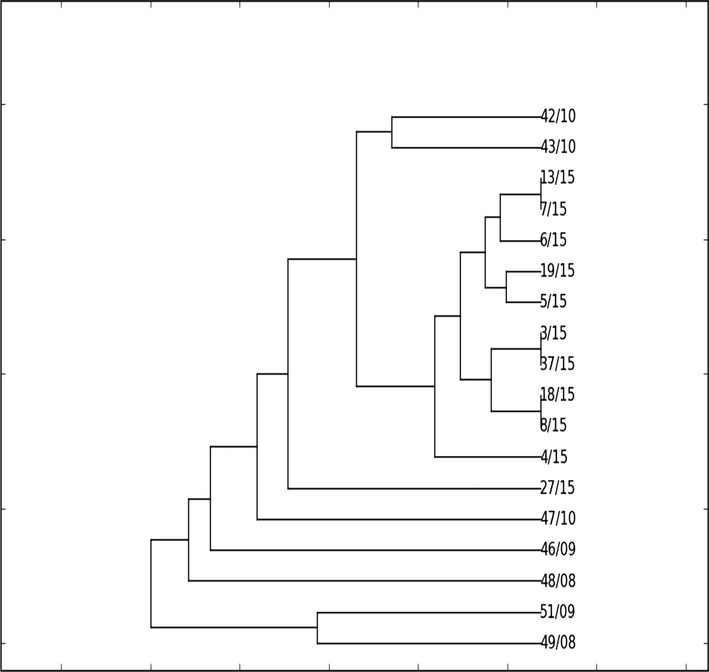
Cluster analysis of *Klebsiella pneumoniae* isolates based on RAPD-PCR typing with the RAPD1 primer

The results from cluster analysis using UPGMA (Fig. 2) showed diversity between strains isolated from 2008 to 2010 and those isolated in 2015. However, relatedness was noted for one *K. pneumoniae* strain isolated in 2008 and another isolated in 2009. Also, two strains isolated in 2010 were also similar in their RAPD profiles. Finally, three separate pairs of *K. pneumoniae* strains isolated in 2015 were found to be closely related based on their genotypic profiles.

## Discussion

The CLSI disk diffusion ESBL confirmatory test proved suitable for the identification of *K. pneumoniae* isolates included in this study, correctly identifying 41 (78.85%) isolates as possessing β-lactamases capable of hydrolysing oximino-cephalosporins. Although it was not the intention of this study to evaluate the effectiveness of the Microscan WalkAway-96 System, the sensitivity being reported here is much less than that reported by Wiegand et al. [15] of 84 and 87% by Vespero et al. [16]. However, this 21.15% (11/52) “false positive” rate should be interpreted with caution as well as interest. In a SENTRY report authored by Bell et al. [17], they found that 20.3% of screen-positive isolates failed to show clavulanate synergy, and, subsequently, 75% of these nonconforming results were due to the presence of a plasmid-borne AmpC enzyme of the CIT or DHA type. Munier et al. [18] also found that 70% of ESBL screen-positive isolates (which were characterised by *E. coli*, *K. pneumoniae*, *K. oxytoca*, and *Proteus mirabilis*) were actually producing an AmpC β-lactamase, while only 13% represented true ESBL producers. Although this study did assess whether isolates that produced negative confirmatory results possessed a gene that coded for an AmpC β-lactamase (data not shown), all of the isolates returned negative results. However, this finding does not rule out the possibility of these isolates harbouring other AmpC varieties, and further investigation is warranted.

Another possible reason that may be posited for the “high” number of false positives involves the influence of the inoculum effect. In their clinical update paper, Patterson and Bonomo highlight that in vitro, MICs of cephalosporins may rise as the inoculum of ESBL-producing organism increases [5]. This was further substantiated by Thauvin-Eliopoulos et al. [19] who showed that the cefotaxime MIC for a *K. pneumoniae* strain harbouring TEM-26 increased from 0.25 μg/ml at an inoculum of 10^5^ CFU/ml to 64 μg/ml at an inoculum of 10^7^ CFU/ml. Similarly, Bedenic et al. [20] found that SHV harbouring *klebsiellae* were more resistant to cephalosporin agents when the inoculum size was higher. This reason may certainly be applicable in the case of this study especially given that eight of nine negative confirmatory test isolates were identified as possessing a SHV-type β-lactamase gene when examined with a molecular assay.

It is also worth noting that even though there was PCR amplification of TEM and SHV genes in most of the isolates, without sequencing it cannot be determined whether these genes contributed to mediating extended spectrum beta-lactam resistance. Sequencing allows for the differentiation of the non-ESBL genes (e.g., TEM-1, -2, and SHV-1) from the ESBL variants (e.g., TEM-3 and SHV-2) [21].

The distribution of the three groups of ESBL-genes for *K. pneumoniae* identified in this study was different from what was reported at this institution by Akpaka et al. [2]. That study reported that there were 84.3% *bla*_TEM_, 34.5% *bla*_SHV_ and 58.8% *bla*_CTX-M_ of the ESBL genes present in *K. pneumoniae* isolates recovered at the institution (EWMSC) over a 3-year period. In comparison, this study found lower rates for *bla*_TEM_ (59%; 39/66) and *bla*_CTX-M_ (46.9%; 31/66, *bla*_CTX-M1_ plus *bla*_CTX-M2_), and a higher rate for *bla*_SHV_ (84.8%; 56/66). Most noteworthy is that 37.8% (25/66) of the *K. pneumoniae* isolates possessed all three β-lactamase genes. This suggests that one or more of these β-lactamase genes may have been acquired from transferrable plasmids, however, a conjugative assay was not performed at the time the study was conducted to confirm this. Moreover, this finding increases the likelihood that other genes such as plasmid-mediated fluoroquinolone and aminoglycoside resistance genes may also be co-transferred, thereby contributing to the dissemination of multidrug resistance mechanisms [22].

The emergence and spread of CTX-M continues to be well documented and reported across Latin America and the Caribbean [2, 9, 23, 24]. In this study there was widespread distribution of β-lactamases belonging to the CTX-M-1 group, and one instance of an isolate with a β-lactamase from the CTX-M-2 group- the first such account of this particular enzyme in *K. pneumoniae* from a Caribbean territory. This simultaneous production of both cefotaximase and ceftazidimase poses a serious problem to the characterization of resistance by clinical laboratories since these enzymes confer a higher level of resistance to oxyimino-cephalosporinases [25].

For RAPD typing, isolates that possessed two or more β-lactamase genes were chosen with the majority of isolates being those recovered in 2015. RAPD-PCR was chosen because it is a rapid and simple method which when optimized has proven competitive with the gold standard of pulse field gel electrophoresis [26, 27]. RAPD1, also referred to as Primer 640 by other authors [13, 26], proved to be optimal for DNA fingerprinting since it allowed the clear distinction of DNA banding patterns for all of the isolates tested. The resulting RAPD profiles for this select group of isolates showed that there was diversity among those isolated during 2008–2010 and those recovered in 2015. However, it was found that *K. pneumoniae* isolates with the same genotype possessed three or more extended-spectrum β-lactamase genes. This seems to suggest that these strains may have been epidemic in the hospital environment, but due to the lack of patient information this fact could not be proven with certainty.

This study was not without some limitations. The loss of transferrable genetic elements, i.e. plasmids, in stored *K. pneumoniae* isolates from 2008 to 2010 could not be accounted for, and, therefore, genes contributing to resistance might have been lost as has been highlighted by previous authors [5]. Secondly, RAPD-PCR was only conducted on a small subset of the isolates recovered due to inadequate resources for DNA extraction and purification.

## Conclusion

This study serves as an important update on the ESBL genes conferring β-lactam antimicrobial resistance among clinical isolates of *K. pneumoniae* isolated from patients being treated at the EWMSC, Trinidad. Along with identifying the traditional *bla*_TEM_, *bla*_SHV_, and *bla*_CTX-M1_ genes, it also identified a previously uncharacterised gene belonging to the *bla*_CTX-M2_ group. These findings suggest that both phenotypic and genotypic methods are required to determine and describe the genes responsible for resistance in *K. pneumoniae*, and thus, better guide infection control and antimicrobial stewardship measures directed at preventing the heterogeneous spread of plasmid-mediated resistance genes. Improving the results of studies, conducted at the EWMSC, similar to ours would require conducting plasmid conjugation transfer experiments and sequencing in order to determine: (1) the extent to which the resistance genes amplified by PCR are chromosomally-mediated or plasmid-mediated, and (2) what ESBL genotypes are prevalent among *K. pneumoniae* and other gram negative bacteria within the hospital setting.

## Additional file

**Additional file 1.** Technical description of the methods used for antimicrobial susceptibility testing and Random amplification of polymorphic DNA.

## Abbreviations

ATCC: American Type Culture
AST: antimicrobial sensitivity testing
*Bla*: used to designate “beta-lactamase” gene
CLSI: Clinical Laboratory Standards Institute
DNA: deoxyribonucleic acid
EWMSC: Eric Williams Medical Sciences Complex
ESBL: extended-spectrum beta (β)-lactamase
ID: identification
MIC: minimum inhibitory concentration
PCR: polymerase chain reaction
RAPD: random amplification of polymorphic DNA
UPGMA: unweighted pair group method with arithmetic averages
