# Supplementary material for: Detection of a CTX-M group 2 beta-lactamase gene in a Klebsiella pneumoniae isolate from a tertiary care hospital, Trinidad and Tobago
Source: Ann Clin Microbiol Antimicrob. 2017 May 8;16:33. doi: 10.1186/s12941-017-0209-x (PMC5421325; doi:10.1186/s12941-017-0209-x)
Supplement: Supplementary file 1 — Additional file 1. Technical description of the methods used for antimicrobial susceptibility testing and Random amplification of polymorphic DNA. [file 12941_2017_209_MOESM1_ESM.docx]

**Technical Description of the method used for Antimicrobial Susceptibility Testing**

After overnight growth of *K. pneumoniae* isolates on Brain Heart Infusion agar (BD), a suspension was made containing the isolates in sterile saline to a turbidity equal to that of a 0.5 McFarland turbidity standard. This was then spread on Mueller-Hinton agar plates by swabbing them with a sterile cotton swab. Thereafter, the antibiotic discs were placed on the plates, incubated 18 to 24 hrs in aerobic conditions at 35°C ± 2°. ESBL production was confirmed if a ≥5-mm increase in a zone diameter for either antimicrobial agent tested in combination with clavulanate vs the zone diameter of the agent when tested alone.

**Technical Description of the method used for Random Amplification of Polymorphic DNA**

The reaction mixture contained 12.5μL GoTaq® Green Master Mix, 5.0μL primer, 2.5μL extract and nuclease free water up to 25μL. Amplification was performed in an Eppendorf Mastercycler Gradient. The reaction mixture was denatured at 94°C for 4 min followed by 50 consecutive cycles of 1 min at 94°C, 2 min at 34°C, and 2 min at 72°C, and a final extension of 10 min at 72°C.
